# Exploration of auditory P50 gating in schizophrenia by way of difference waves

**DOI:** 10.1186/1744-9081-2-6

**Published:** 2006-01-28

**Authors:** Sidse M Arnfred

**Affiliations:** 1 Experiments performed at the Department of Psychiatry, Bispebjerg Hospital, University Hospital of Copenhagen, Bispebjerg Bakke 23, DK-2400 København NV, Denmark

## Abstract

Electroencephalographic measures of information processing encompass both mid-latency evoked potentials like the pre-attentive auditory P50 potential and a host of later more cognitive components like P300 and N400.

Difference waves have mostly been employed in studies of later event related potentials but here this method along with low frequency filtering is applied exploratory on auditory P50 gating data, previously analyzed in the standard format (reported in *Am J Psychiatry *2003, **160**:2236-8). The exploration was motivated by the observation during visual peak detection that the AEP waveform was different in the patient group, although this was not reflected by the peak measures. The sample included un-medicated schizophrenia spectrum patients (n = 17) and healthy controls (n = 24). The patients had an attenuated difference P50. This attenuation was primarily seen in the sub-sample of patients with severe negative symptoms. The difference attenuation was due to low amplitude at the first stimulus. This suggests an abnormality in readiness more than an abnormality in gating in the patient group.

## Introduction

The deficiency in auditory P50 gating reported in numerous studies of schizophrenic patients has been one of the experimental findings that have supported the theory of defect sensory gating in schizophrenia [1]. P50 gating is the relative amplitude reduction of auditory evoked potential (AEP) P50 from the first stimulus (S1) to the second stimulus (S2). P50 is the second positive component of the mid-latency AEP; a scalp electroencephalographic measure of the brain response to auditory stimulation. The P50 paradigm consists of two identical clicks presented with an interval of 0.5 s followed by a 8–12 s pause before the next paired stimulation.

While P50 gating mostly has been reported as a ratio measure (S2/S1 or 1- S2/S1 × 100), it has been suggested that a difference measure might be more reliable [2]. The latter report suggested the use of the traditional peak amplitudes for the difference measures. Extending this idea to the whole waveform, subtraction waveforms also called difference waves are investigated presently. Difference waves, where the evoked potential of the same individual recorded under one condition is subtracted from a potential recorded in another condition, have been used particularly in the research of the Mis Match Negativity (MMN). The MMN is a negative potential shift evoked when a deviant stimulus is presented in a series of well-known stimuli. It is observed even when attention is focused on something else than the stimulus series, and consequently MMN has been conceived as a manifestation of involuntary attention and as such part of the orienting response [3]. P50 is at a shorter latency than the MMN but it has been demonstrated that attentional modulation of the AEP starts at 15 ms post stimulus [4]. In this sense an abnormal P50 difference wave in schizophrenia might be an indication of faulty involuntary attentional processing at the same time as a small or negative difference wave could imply a gating defect.

The present data were recorded as part of a larger study examining gating in un-medicated schizophrenia spectrum patients and data on subsamples of the present patients have been reported previously [5,6]. It is noteworthy that auditory P50 gating following the standard processing method [7] and two other comparisons methods also involving the ratio measure was normal in the schizophrenic patients included in the present sample [5]. However, when performing the visual peak detection on the P50 gating data, it was observed that the AEP waveform was different in the 30–70 ms latency range in the patient group, although this was not reflected by the peak measures. Thus, this is an explorative investigation of using difference waves on the mid-latency auditory evoked potential recorded in a P50 gating paradigm and correlating this to symptom measures.

## Methods

Patients (n = 17) were included if they had a diagnosis of schizophrenia spectrum disorder, age 18 to 50 years, no current medication and no substance abuse except tobacco. The controls (n = 24) were physical and mentally healthy men of ages that matched the patients. All subjects gave informed consent as approved by the Ethics Committee and they were paid to participate in the experiment. Only men were included due to large gender variation on other measures of interest than the present. DSM-IV diagnoses were established by the Schedules for Assesment of Neuropsychiatry (SCAN) version 2.1 [8]. The Schedules for Assesment of Positive Symptoms of Schizophrenia (SAPS) [9] and Schedules for Assesment of Negative Symptoms of Schizophrenia (SANS) [10] were performed regarding the last three months. Five patients had schizotypal personality disorder and twelve patients were schizophrenic. Psychopathology and other parameters of illness is tabulated in table 1, where the sample is stratified by their negative symptom score (see below). Among the patients 12 subjects and among comparison subjects 9 subjects smoked more than 10 cigarettes a day. Details of smoking habits are given in table 2.

**Table 1:** Amplitudes of mid-latency auditory gating difference wave, P50 gating ratio, sweeps included in the EPs and tobacco consumption in schizophrenia spectrum patients (stratified by their negative symptom score) and healthy controls

	***CON***	***SCH ***	***SCH low***	***SCHhigh***
**Onset**				
μV				
Fz	0.40**	-1.30	-0.68	-1.98
	*1.41*	*1.68*	*1.42*	*1.77*
Cz	1.13**	-0.37	0.32	-1.14
	*1.35*	*1.14*	*1.91*	*2.24*
Pz	0.62	-0.12	0.88*	-1.25
	*1.50*	*2.13*	*1.26*	*2.41*
Fp1	-0.32*	-1.27	-0.59	-2.03
	*1.30*	*1.28*	*0.81*	*1.31*
Fp2	-0.35*	-1.37	-0.59*	-2.24
	*1.30*	*1.44*	*0.87*	*1.49*
C3'	0.33	-0.17	0.33	-0.74
	*0.90*	*1.68*	*1.32*	*1.94*
C4'	0.57	-0.01	0.46	-0.53
	*0.99*	*1.29*	*1.21*	*1.25*

**Peak**				
μV				
Fz	1.31**	-0.06	0.85*	-1.08
	*1.64*	*1.71*	*0.91*	*1.87*
Cz	1.90*	0.44	1.21	-0.42
	*1.64*	*1.58*	*1.95*	*2.56*
Pz	0.65	-0.30	0.56*	-1.28

	*1.31*	*1.71*	*1.19*	*2.29*
Fp1	0.13	-0.41	0.58**	-1.52
	*1.26*	*1.58*	*1.04*	*1.35*
Fp2	0.20	-0.40	0.77**	-1.71
	*1.38*	*1.82*	*1.27*	*1.43*
C3'	0.76	0.11	0.44	-0.26
	*1.04*	*1.26*	*1.17*	*1.34*
C4'	0.95	0.22	0.76	-0.39
	*0.82*	*1.59*	*1.26*	*1.78*

***Other Group Variables***				
St. P50rat	0.40	0.35	0.35	0.35
	*0.30*	*0.22*	*0.21*	*0.25*
S1Epochs	98**	77	83	71
	*12*	*23*	*18*	*28*
S2Epochs	101**	85	94	76
	*12*	*23*	*16*	*26*
Tobacco	34	75	108	38
	*47*	*73*	*66*	*63*

Mean amplitudes in μV (*standard deviation*). *) p < 0.05; **) p < 0.01 (post-hoc pairwise comparisons, Bonferroni corrected) when in (***CON***) denoting probability of significant difference between healthy controls (***CON***, N = 24) and all the schizophrenia spectrum patients (***SCH***, N = 17), when in (***SCHlow***) denoting probability of significant difference between patients having a negative symptom score (sum of five global SANS (not including global attention) items) ranging 0–7 (***SCHlow***, N = 9) and the patients having scores ranging 8–12 (***SCHhigh***, N = 8). The P50 ratio (S2/S1 amplitude) following traditional 10–50 Hz digital filtering has previously been reported [5], where it is compared to two other types of digital filtering. Number of included sweeps in the EP in both stimuli (S1 and S2) differs between healthy comparisons subjects and patients, but not within the patient sample.

**Table 2:** Illness parameters in the patient group stratified by the negative symptom score

	***SCH low***	***SCHhigh***
Positive symptoms	4.0	3.8
	*2.6*	*1.8*
Disorganized	2.9	2.1
symptoms	*2.8*	*2.4*
Negative symptoms	3.9**	10.1
	*2.6*	*1.5*
Time since medication	76	29
(months) ^#^	*88*	*29*
	(N = 5)	(N = 8)
Total duration of med.	5.2	8.1
treatment (months)	*8.8*	*8.7*
Duration of illness	6.7	8.5
(years)	*7.9*	*8.5*

Mean values and *standard deviations*. ***SCHhigh***: N = 8 ; ***SCHlow***: N = 9, apart from #) "Time since medication", as four patients were drug-naïve. The SAPS and SANS scores are given within three dimensions of schizophrenia psychopathology: Positive (global hallucinations + global delusions), disorganised symptomatology (global Formal thought disorder + global bizarre behavior + SANS item inappropriate affect) and negative (all global scores of the SANS except attention). The negative score stratified the sample. "Duration of illness" is measured as time since first psychiatric contact. **) p < 0.01. Two of the non-psychotic patients were in the ***SCHhigh ***group. In the ***SCHhigh ***group three patients had only received traditional type neuroleptics, one had only had modern antipsychotics and one patient had tried both types of treatment. In the ***SCHlow ***group two patients had received traditional type neuroleptics, and six had received modern type antipsychotics.

Recording and stimulus equipment and settings and the seven electrode montage (Fp1, Fp2, Fz, C3', C4', and Pz + EOG) were identical to earlier reports from our laboratory [11]. Auditory stimuli were clicks of 20–10000 Hz, 1.6 ms duration and 104 dB peSPL delivered binaurally through earphones. During recording the subjects were seated comfortably upright with closed eyes in dim light and with background masking low level (70 dB SPL) white noise. The subject was enrolled in a fixed schedule of several types of EEG experiments and breaks that included two hourly recording sessions before lunch and one hourly session after lunch. In each hourly session one run was recorded of the auditory gating paradigm of 40 paired click stimuli. Sampling rate was 1 kHz pr channel. Subjects were allowed to smoke in the fixed breaks but not the last 15 minutes before resuming recording. Sweeps were rejected if the EOG amplitude exceeded +/- 70 μV, but no baseline correction was performed. As the low frequency activity was examined it was considered important to avoid the de-trending, which would be the consequence of correcting with a baseline mean measured across a slow wave. The possible confounding effect of a difference in baseline was examined by an analysis of variance (ANOVA), as described below, of the maximum value in the latency range -5 to +5 ms. No group differences were observed in baseline or in EOG amplitudes. Following initial exploration and recent results of selective low pass filtering [12] the digital frequency band-pass was set at 1–15 Hz (24 dB/oct roll-off). The difference waveform was computed by point-to-point subtraction of the S2 waveform from the S1 waveform. P50d onset (minimum amplitude in the latency range 30–50 ms, leading electrode Fz) and P50d peak (max amplitude in the latency range 40–80 ms, leading electrode Fz) were based on computerised detection. The difference amplitudes were analysed using repeated measures analyses of variance (r.m. ANOVA) in an Electrode*Group matrix. Exploratory, the different parameters of illness as listed in table 1 was entered the analysis as covariates. Only negative symptoms covaried with P50d onset (F_*1,15 *_: 5.413, p = .03). Consequently, the patient group was stratified by the median of their negative symptom score. Post-hoc one-way ANOVA was performed for each channel. One-way ANOVAs were also performed for the standard P50 gating measure, previously reported [5], the number of sweeps included, and the number of cigarettes pr week as listed in table 2. The reported p-values are Bonferroni corrected for multiple comparions.

## Results

**Figure 1:**
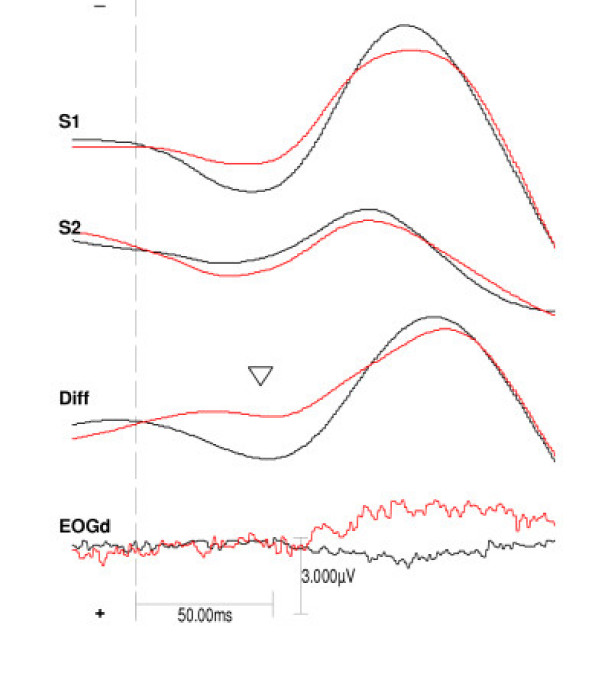
**Grand average of stimulus one, stimulus two and difference wave at the central frontal channel in the two groups**. The grand average auditory evoked potentials at the frontal channel (Fz) in the control group (black line, N = 24) and the schizophrenia spectrum patients (red line, N = 17). From top the stimulus one (S1) waveform, the stimulus two (S2) waveform, and the difference wave (S1–S2) in the groups. At bottom the difference wave of the electro-oculogram (EOGd) Arrowhead points at P50d.

The patients had more negative difference amplitudes than the controls in P50d onset (main effect of group F _1,39 _= 7.925, p = .008) and in P50d peak (main effect of group F _1,39 _= 5.811, p = .02). See figure 1 and table 1. The same pattern was seen when analysing the S1 P50 onset amplitude (main effect of group F _1,39 _= 6.738, p = .01) but only as a trend in S1 P50 peak (main effect of group F _1,39 _= 3.314, p = .08). No group differences were seen at the S2 amplitudes. The most negative amplitudes were seen in the patients having high ratings on the SANS (P50d onset: F _1,15 _= 6.290, p = .02; P50d peak: F _1,15 _= 10.081, p = .006).

The AEPs were derived from more epochs in the healthy comparison group than the patient group, but no difference was observed between the patient subgroups, see table 2.

## Discussion

The patients had a negative difference amplitude in the 30–70 ms latency range frontally, while the healthy controls had a positive P50 difference component, a finding that is in agreement with most previous studies of P50 gating in schizophrenia based on 10–50 Hz filtered data and the ratio measure [7,13]. The major group difference of the P50d onset amplitude was also seen in the S1 amplitude but not in the S2 amplitude. This finding is in agreement with a study by [12], in which the low frequency P50 S1 amplitude was the group discriminating factor.

The patients having high negative symptom scores had a particular augmentation of negative amplitude at the prefrontal channels, corresponding to a lack of positive deflection in S1 at vertex. The possibility of contamination by reflex eye movements in S1 is contradicted by the lack of correlation to the number of rejected sweeps. Contamination by volume conduction from the frontalis muscle EMG would mostly be filtered out by the frequency band selected, but it cannot be ruled out [14]. Then the findings would have to be interpreted as increased reflex activity with increasing negative symptoms an effect which could be related to an orienting response or a slight startle reflex. The orienting response has been extensively investigated in schizophrenia by way of the skin conductance response reviewed in [15-17]. Increased negative symptoms are associated with decreased orienting response and this is not in accordance with the findings here [18]. Startle habituation is decreased in schizophrenic patients, but it is not correlated to negative symptoms [19]. Recording of P50 gating and simultaneous facial muscle activity in schizophrenic patients would be necessary to solve the issue.

Measuring P50 gating as ratio, one of the earlier reports showed no difference in gating between schizophrenic patients low and high in negative symptoms [20]. Later studies have mostly supported this. Light and colleagues [21] reported that negative symptoms only accounted for 2% of the variance in gating and a recent meta-analysis showed that symptoms do not predict P50 gating, although this might be due to insuffient statistical power [22]. In opposition to this but in agreement with the present low frequency difference analysis of P50 data, Ringel and colleagues found a positive correlation between the negative symptom subscale of the Positive and Negative Syndrome Scale for Schizophrenia (PANSS) and gating deficits [23]. The negative items on SANS and PANSS have highly significant correlations [24]. Furthermore, Yee and colleagues [13] found that anergia and attentional impairment correlated with the gating deficiency. In the present data the ratio measure of P50 gating did not show any difference between the two groups nor between patient subgroups [5]. This could imply that the low frequency filtering unveil differences between patient subgroups particularly on S1 in accordance with several studies where the main difference in gating has been explained by variations in the S1 amplitude [25-29].

The P50 paradigm was at first reported to only track so-called "automatic" or pre-attentive processing [1,26,30,31]. Nonetheless, later studies of P50 [32,33] showed a direct effect of task allocation on the S1 and S2 amplitudes. Amplitude increase was seen when a discrimination task was on either S1 [31,7] or S2 [31,32,34]. When subjects were distracted during recording S1 amplitude was attenuated and S2 amplitude constant [31,7]. It seems possible that the low frequency S1 P50 amplitude tracks an aspect of involuntary attention, which are delayed and diminished in schizophrenic patients. Several theories concerning the basic deficits in schizophrenia exist. Among them, a hypothesized weakening of the effect of regularity on stimulus processing [[35], [36], [37]] i.e. a decrease in expectancy during repeated stimulation seems to fit the present finding of low S1 amplitude best.

In conclusion, un-medicated male schizophrenia spectrum patients show an attenuation of low frequency amplitude at the first stimulus of the P50-gating paradigm. This is likely to reflect an abnormality in readiness and not an abnormality in gating.

## Competing interests

The auhtor(s) declare that they have no competing intersts.

## References

[B1] Braff DL (1993). Information processing and attention dysfunctions in schizophrenia. Schizophr Bull.

[B2] Smith DA, Boutros NN, Schwarzkopf SB (1994). Reliability of P50 auditory event-related potential indices of sensory gating. Psychophysiology.

[B3] Naatanen R, Simpson M, Loveless NE (1982). Stimulus deviance and evoked potentials. Biol Psychol.

[B4] Hackley SA (1993). An evaluation of the automaticity of sensory processing using event- related potentials and brain-stem reflexes. Psychophysiology.

[B5] Arnfred SM, Chen AC, Glenthoj BY, Hemmingsen RP (2003). Normal p50 gating in unmedicated schizophrenia outpatients. Am J Psychiatry.

[B6] Arnfred SM, Chen AC (2004). Exploration of somatosensory P50 gating in schizophrenia spectrum patients: reduced P50 amplitude correlates to social anhedonia. Psychiatry Res.

[B7] White PM, Yee CM (1997). Effects of attentional and stressor manipulations on the P50 gating response. Psychophysiology.

[B8] Wing JK, Sartorius N, Ûstün TB (1998). Diagnosis and clinical measurement in psychiatry.  A reference manual for SCAN.

[B9] Andreasen N (1984). Scale for Assessment of Positive Symptoms (SAPS).

[B10] Andreasen N (1984). Scale for Assessment of Negative Symptoms (SANS).

[B11] Arnfred SM, Eder DN, Hemmingsen RP, Glenthoj BY, Chen AC (2001). Gating of the vertex somatosensory and auditory evoked potential P50 and the correlation to skin conductance orienting response in healthy men. Psychiatry Res.

[B12] Clementz BA, Blumenfeld LD (2001). Multichannel electroencephalographic assessment of auditory evoked response suppression in schizophrenia. Exp Brain Res.

[B13] Yee CM, Nuechterlein KH, Morris SE, White PM (1998). P50 suppression in recent-onset schizophrenia: clinical correlates and risperidone effects. J Abnorm Psychol.

[B14] Perlstein WM, Simons RF, Graham FK (2001). Prepulse effects as a function of cortical projection system. Biol Psychol.

[B15] Bernstein AS, Schnur DB, Bernstein P, Yeager A, Wrable J, Smith S (1995). Differing patterns of electrodermal and finger pulse responsivity in schizophrenia and depression. Psychol Med.

[B16] Bernstein AS, Frith CD, Gruzelier JH, Patterson T, Straube E, Venables PH, Zahn TP (1982). An analysis of the skin conductance orienting response in samples of American, British, and German schizophrenics. Biol Psychol.

[B17] Dawson ME, Nuechterlein KH (1984). Psychophysiological dysfunctions in the developmental course of schizophrenic disorders. Schizophr Bull.

[B18] Bernstein AS (1987). Orienting response research in schizophrenia: where we have come and where we might go. Schizophr Bull.

[B19] Parwani A, Duncan EJ, Bartlett E, Madonick SH, Efferen TR, Rajan R, Sanfilipo M, Chappell PB, Chakravorty S, Gonzenbach S, Ko GN, Rotrosen JP (2000). Impaired prepulse inhibition of acoustic startle in schizophrenia. Biol Psychiatry.

[B20] Adler LE, Waldo MC, Tatcher A, Cawthra E, Baker N, Freedman R (1990). Lack of relationship of auditory gating defects to negative symptoms in schizophrenia. Schizophr Res.

[B21] Light GA, Geyer MA, Clementz BA, Cadenhead KS, Braff DL (2000). Normal P50 suppression in schizophrenia patients treated with atypical antipsychotic medications. Am J Psychiatry.

[B22] Bramon E, Rabe-Hesketh S, Sham P, Murray RM, Frangou S (2004). Meta-analysis of the P300 and P50 waveforms in schizophrenia. Schizophr Res.

[B23] Ringel TM, Heidrich A, Jacob CP, Pfuhlmann B, Stoeber G, Fallgatter AJ (2004). Sensory gating deficit in a subtype of chronic schizophrenic patients. Psychiatry Res.

[B24] Kay SR, Opler LA (1987). The positive-negative dimension in schizophrenia: its validity and significance. Psychiatr Dev.

[B25] Judd LL, McAdams L, Budnick B, Braff DL (1992). Sensory gating deficits in schizophrenia: new results. Am J Psychiatry.

[B26] Freedman R, Adler LE, Gerhardt GA, Waldo M, Baker N, Rose GM, Drebing C, Nagamoto H, Bickford-Wimer P, Franks R (1987). Neurobiological studies of sensory gating in schizophrenia. Schizophr Bull.

[B27] Jin Y, Potkin SG, Patterson JV, Sandman CA, Hetrick WP, Bunney WEJ (1997). Effects of P50 temporal variability on sensory gating in schizophrenia. Psychiatry Res.

[B28] Jin Y, Bunney WEJ, Sandman CA, Patterson JV, Fleming K, Moenter JR, Kalali AH, Hetrick WP, Potkin SG (1998). Is P50 suppression a measure of sensory gating in schizophrenia?. Biol Psychiatry.

[B29] Patterson JV, Jin Y, Gierczak M, Hetrick WP, Potkin S, Bunney WEJ, Sandman CA (2000). Effects of temporal variability on p50 and the gating ratio in schizophrenia: a frequency domain adaptive filter single-trial analysis. Arch Gen Psychiatry.

[B30] Freedman R, Adler LE, Waldo MC, Pachtman E, Franks RD (1983). Neurophysiological evidence for a defect in inhibitory pathways in schizophrenia: comparison of medicated and drug-free patients. Biol Psychiatry.

[B31] Jerger K, Biggins C, Fein G (1992). P50 suppression is not affected by attentional manipulations. Biol Psychiatry.

[B32] Guterman Y, Josiassen RC (1994). Sensory gating deviance in schizophrenia in the context of task related effects. Int J Psychophysiol.

[B33] Jin Y, Potkin SG (1996). P50 changes with visual interference in normal subjects: a sensory distraction model for schizophrenia. Clin Electroencephalogr.

[B34] Guterman Y, Josiassen RC, Bashore TRJ (1992). Attentional influence on the P50 component of the auditory event- related brain potential. Int J Psychophysiol.

[B35] Hemsley DR (1996). Schizophrenia. A cognitive model and its implications for psychological intervention. Behav Modif.

[B36] Hemsley DR (1993). A simple (or simplistic?) cognitive model for schizophrenia. Behav Res Ther.

[B37] Shakow D (1971). Some observations on the psychology (and some fewer, on the biology) of schizophrenia. J Nerv Ment Dis.

